# The quantitative haemodynamic effect of levosimendan, dobutamine, and milrinone in heart failure patients: a meta-analysis

**DOI:** 10.1093/eschf/xvag120

**Published:** 2026-04-24

**Authors:** Vincenzo Nuzzi, Cristina Madaudo, Paolo Manca, Antonio Cannata, Giuseppe Raffa, Massimiliano Mulè, Stefano Cannata, Eluisa La Franca, Calogera Pisano, Manlio Cipriani, Roberto Lorusso

**Affiliations:** Clinical Cardiology and Heart Failure Unit, IRCCS-ISMETT (Mediterranean Institute for Transplantation and Specialized Therapies), Via Tricomi 5, Palermo 90127, Italy; Cardiothoracic Surgery Department, Heart and Vascular Centre, Maastricht University Medical Centre, Cardiovascular Research Institute Maastricht (CARIM), Maastricht, The Netherlands; Department of Health Promotion, Mother and Child Care, Internal Medicine and Medical Specialties (ProMISE) University of Palermo, Italy; Clinical Cardiology and Heart Failure Unit, IRCCS-ISMETT (Mediterranean Institute for Transplantation and Specialized Therapies), Via Tricomi 5, Palermo 90127, Italy; School of Cardiovascular Medicine and Sciences, King's College London, London, UK; IRCCS-ISMETT (Mediterranean Institute for Transplantation and Specialized Therapies), Palermo, Italy; Department of Precision Medicine in Medical Surgical and Critical Area (Me.Pre.C.C.), University of Palermo, Palermo 90134, Italy; Clinical Cardiology and Heart Failure Unit, IRCCS-ISMETT (Mediterranean Institute for Transplantation and Specialized Therapies), Via Tricomi 5, Palermo 90127, Italy; Unit of Interventional Cardiology, IRCCS-ISMETT (Mediterranean Institute for Transplantation and Specialized Therapies), Palermo, Italy; Clinical Cardiology and Heart Failure Unit, IRCCS-ISMETT (Mediterranean Institute for Transplantation and Specialized Therapies), Via Tricomi 5, Palermo 90127, Italy; IRCCS-ISMETT (Mediterranean Institute for Transplantation and Specialized Therapies), Palermo, Italy; Department of Precision Medicine in Medical Surgical and Critical Area (Me.Pre.C.C.), University of Palermo, Palermo 90134, Italy; Clinical Cardiology and Heart Failure Unit, IRCCS-ISMETT (Mediterranean Institute for Transplantation and Specialized Therapies), Via Tricomi 5, Palermo 90127, Italy; Cardiothoracic Surgery Department, Heart and Vascular Centre, Maastricht University Medical Centre, Cardiovascular Research Institute Maastricht (CARIM), Maastricht, The Netherlands

**Keywords:** Inotropes, Heart failure, Invasive haemodynamic, Advanced heart failure, Meta-analysis

## Abstract

**Introduction:**

Inotropic therapy is a cornerstone of medical treatment for patients with low-output heart failure (HF). We aimed to investigate the quantitative effect of specific inotropic drugs on invasive haemodynamics.

**Methods:**

This meta-analysis assessed the haemodynamic effects of dobutamine, levosimendan, and milrinone in patients with low-output HF. Only studies using invasive haemodynamic assessment were included. The primary outcome was the quantitative change in variables such as cardiac index, pulmonary artery wedge pressure, mean pulmonary artery pressure, mean arterial pressure (MAP), pulmonary and systemic vascular resistance (before and after administration of each drug. Quality was assessed using the National Institutes of Health Quality Assessment Tool (NIH-QAT). A sensitivity analysis compared the effect on acute versus chronic HF populations.

**Results:**

Twenty-six studies (*n* = 1888 patients) were included in the analyses. Based on the NIH-QAT checklist, 11 studies were at low risk of bias, 14 at moderate risk, and 1 at high risk. Meta-analysis showed that all the study drugs improved the haemodynamic variables assessed, without significant differences amongst them, except for MAP (*P* = .0486). Dobutamine and levosimendan caused a non-significant increase in MAP, while milrinone showed a trend towards a reduction in MAP [−3.46 (−7.27 to +0.35)]. The heterogeneity across studies was high. In the sensitivity analysis, dobutamine improved CI more than levosimendan in patients with chronic HF.

**Conclusion:**

The use of inotropes improves haemodynamic status in patients with low-output HF, with no consistent superiority of one agent over the others. These findings support the current clinical practice of agent selection based on individual patient characteristics. Head-to-head trials in well-phenotyped HF populations are warranted to guide personalized inotrope use.

## Graphical Abstract

**Graphical Abstract:**
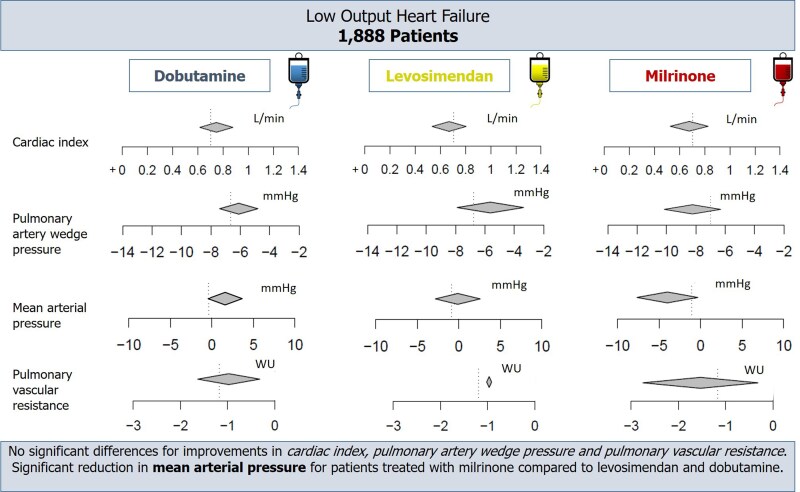
Forest plot showing the pooled effects of each drug across all included studies on the invasive haemodynamic variables analysed. Dobutamine is shown on the left, levosimendan in the centre, and milrinone on the right.

## Introduction

The medical treatment of patients with acute heart failure (HF) remains challenging with high mortality, and several clinical trials over the past decades have failed to demonstrate a survival benefit with any pharmacological intervention.^1^ Indeed, while the prognosis of patients with chronic HF has recently markedly improved, the risk of in-hospital death following a hospitalization for acute HF has been unchanged in the last two decades.^2–4^ Clinical trials in this setting are particularly difficult to design, due to the marked heterogeneity of acute HF populations, challenging adherence to research protocols, including inclusion and exclusion criteria, in the acute phase and high risk of competing events.

The use of inotropes is widespread worldwide, although the evidence supporting a prognostic benefit from these drugs is still a matter of debate.^5^ Patients with acute HF often present in a low-output state leading to significant hypoperfusion of vital organs, including the heart itself, and consequent multiorgan failure.^6^ Similarly, vasodilators have a clear pathophysiological rationale in this setting, but their impact on long-term outcomes remains uncertain, highlighting the limited evidence base for both therapeutic approaches.^7,8^

Inotropic therapy plays a pivotal role even in patients with chronic advanced HF. Indeed, haemodynamic optimization is essential in those being evaluated for advanced therapies, such as durable ventricular assist device implantation or heart transplantation.^9,10^ Also in this setting, selecting the appropriate inotropic agent remains clinically relevant and challenging.

Several mechanisms have been proposed to explain both the benefits and the harms of inotropes. The primary mechanism underlying their potential benefit is the haemodynamic improvement. Different inotropes rely on different mechanisms of action and might exert different effects. The first inotropes used in clinical practice (dobutamine, dopamine, adrenaline) act via beta-adrenergic receptor stimulation on the cardiomyocytes leading to increased intracellular calcium concentration. On the other hand, inotropes such as levosimendan and milrinone act differently, enhancing cardiac contractility by increasing calcium sensitivity or by inhibiting phosphodiesterase III, respectively.^11^ The magnitude of increase in cardiac function depends on the specific characteristics of each drug and on the dose administered. Understanding the expected effect on cardiac output, peripheral perfusion, cardiac filling pressures, systemic (SVR), and pulmonary vascular resistance (PVR) and circulation is crucial to individualize inotrope therapy. In addition, evidence on differences, or equivalences, amongst agents could help clinicians in the choice of the optimal drug according to other established characteristics (i.e. duration of the effect, contraindications, tolerability, costs, and availability). Therefore, we conducted a meta-analysis aiming to quantify the haemodynamic effect of dobutamine, levosimendan, and milrinone in patients with HF and low-output state.

## Methods

### Protocol, search strategy, and outcomes

**Figure 1:**
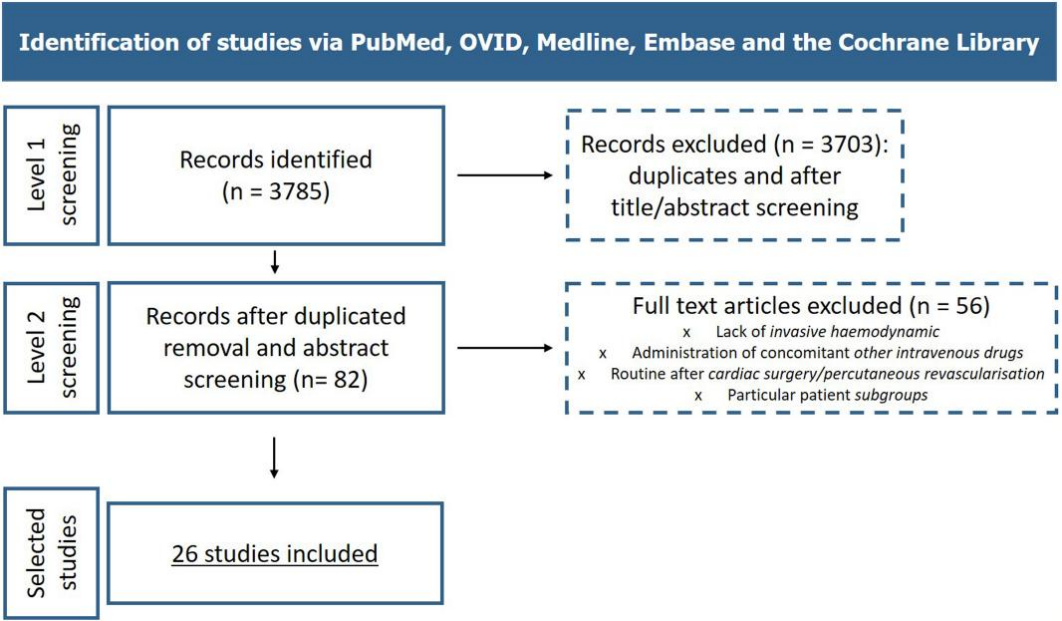
Flowchart of the selection process for the included studies

This meta-analysis was performed in accordance with the Preferred Reporting Items for Systematic Reviews and Meta-Analysis (PRISMA) guidelines. In line with PRISMA, a PICO strategy was followed (*Table 1*). We focused on studies evaluating the acute haemodynamic effect of dobutamine, levosimendan and milrinone in an in-hospital setting in patients with acute or chronic low-output HF and reduced ejection fraction. All the patients had an indication for inotrope therapy and, therefore, were severely symptomatic (New York Heart Association class IV). The decision to focus our analyses on these drugs was based on preliminary literature research which showed that dobutamine, levosimendan and milrinone had an appropriate number of studies available. We included only studies evaluating haemodynamic variables through invasive measurement, by Swan-Ganz catheterization or right heart catheterization in the cathlab. The variables analysed were: cardiac index (CI), pulmonary artery mean pressure (PAPm), mean arterial pressure (MAP), pulmonary artery wedge pressure (PAWP), PVR, and SVR. Studies in which patients clearly received concomitant other intravenous drugs that could influence haemodynamic data were excluded. Similarly, we excluded studies on routine administration of the study drug before or after cardiac surgery/percutaneous revascularization, as well as those enrolling peculiar patient’ subgroups. The outcome was the quantitative within-patient change in haemodynamic variables following initiation of the study drug. When multiple haemodynamic follow-up measurements were available, the 24-hour measurement was preferred. Both observational studies and randomized clinical trials were included, but only data from patients receiving an active treatment were considered. Exclusion criteria for article selection included the following: articles not in English, review articles, meta-analyses, duplicates, unpublished data, abstracts and non-peer-reviewed articles. Only studies involving humans were considered to empower clinical relevance and applicability. A researcher (V.N.) systematically searched PubMed, OVID, Medline, Embase, and the Cochrane Library for publications before 1 February 2025. Predefined keywords and Medical Subject Headings (MeSH), including ‘heart failure’, ‘cardiogenic shock’, ‘cardiac failure’, ‘observational study’, ‘cohort study’, ‘case-control’, ‘randomised clinical trial’, ‘right heart catheterisation’, ‘cardiac index’, ‘wedge pressure’, ‘inotrope’, ‘dobutamine’, ‘milrinone’, ‘levosimendan’ and all their synonyms (Supplementary Table S1) were used. Two researchers (V.N. and C.M.) independently screened the titles and abstracts of all identified studies, selecting those of potential interest (level 1 screening). Thereafter, they examined the full text of the selected papers to finalize the selection of the study (level 2 screening) (*Figure 1*). Discrepancies were shared and resolved by consensus with a third reviewer (A.C.). The data extracted included: first author’s name, year of publication, country, study design, patients included (acute or chronic HF), study drug administered and haemodynamic outcomes. This study did not require ethical approval or patient consent. The research protocol was registered on PROSPERO (ID: 1133068).

**Table 1:** PICOS (Population, Intervention, Comparison, Outcomes, and Study) data for formulating eligibility criteria in the meta-analysis

PICOS (Population, Intervention, Comparison, Outcomes, and Study) data for formulating eligibility criteria in the meta-analysis	
Population	Patients with heart failure and low-output
Intervention	Inotrope administration (dobutamine or levosimendan or milrinone)
Comparator	Haemodynamic status before the intervention
Outcomes	Haemodynamic improvement assessed by right heart catheterization
Setting	All Study Settings

### Quality assessment

Two researchers (V.N. and C.M.) independently evaluated the quality of the included studies using the National Institutes of Health Quality Assessment Tool (NIH-QAT), comprising 12 items. Any inconsistencies or doubts were reviewed by a third researcher (A.C.). Each study was categorized as low risk of bias (9–10 criteria met), moderate risk of bias (7–8 criteria met), or high risk of bias (<5 criteria met).

### Statistical analysis

For each haemodynamic parameter (MAP, CI, mPAP, WP, PVR, SVR), the mean difference (MD) between post- and pre-treatment values was calculated for each study arm. For each haemodynamic parameter (MAP, CI, mPAP, PAWP, PVR, SVR), the MD between post- and pre-treatment values was calculated for each study arm. For paired pre–post data, the standard deviation (SD) of the change was derived from the pre- and post-treatment variability, assuming a within-patient correlation coefficient of 0.5, in line with previous meta-analyses of haemodynamic pre–post data. The standard error of the MD was then obtained by dividing the SD of the change by the square root of the sample size. Random-effects models were employed for all meta-analyses, considering the expected clinical and methodological heterogeneity across studies. Between-study heterogeneity was quantified using the *I*^2^ statistic and *τ*^2^, with *I*^2^ values of 30%, 50%, and 75% interpreted as low, moderate, and high heterogeneity, respectively. Subgroup analyses were conducted by drug class (Milrinone, Levosimendan, Dobutamine) and by study setting (acute vs chronic). Differences between subgroups were tested using *χ*^2^ statistics. We performed additional exploratory analyses including random-effects meta-regression (REML) using available study-level covariates (baseline haemodynamic values, acute vs chronic setting, study design, inotrope dose and clinical setting), as well as influence diagnostics (studentized residuals, hat values, DFBETAs and Cook’s distances), Baujat plots and leave-one-out analyses to identify influential studies. A value of *P* < .05 was considered significant. All analyses were performed with R statistical package version 4.2.2 (R Foundation).

### Sensitivity analysis

To assess the robustness of our findings, multiple sensitivity analyses were performed. First, we stratified studies according to clinical setting (‘acute’ vs ‘chronic’ HF) to explore whether haemodynamic responses differed across clinical presentations. The direction and magnitude of pooled effects were consistent across settings. Second, we conducted analyses stratified by study design, comparing randomized controlled trials (RCTs) with observational studies. Despite expected differences in populations and methodological rigour, pooled haemodynamic effects remained broadly comparable, with overlapping confidence intervals and no significant subgroup interaction under the random-effects model. Third, because inotropic effects may vary by drug exposure, we performed an additional sensitivity analysis restricted to studies with clearly reported dosing regimens (including dose, bolus use, and infusion duration). Across all haemodynamic endpoints, effect estimates in this restricted dataset closely matched those of the full analysis, with minimal changes in pooled MDs and preserved directionality. Finally, studies with missing or inconsistent information required to compute standard errors (e.g. SDs or sample size) were excluded from sensitivity analyses to minimize imprecision. Overall, all sensitivity analyses confirmed the stability of the main findings, indicating that the observed haemodynamic effects were not driven by clinical setting, study design, or dosing inconsistencies.

### Assessment of publication bias

Potential publication bias was evaluated for each of the six haemodynamic outcomes (CI, MAP, mPAP, PAWP, PVR and SVR). For each endpoint, we generate a dedicated funnel plot and assessed small-study effects using Egger’s regression test, applied to meta-analyses including ≥3 studies. Funnel plot asymmetry was visually inspected, and Egger’s test. *P*-values were used as a formal statistical measure. Analyses were performed under the same random-effects framework used for the primary models.

## Results

### Study selection

The initial literature research shortlisted 3785 abstracts. After removal of duplicates and screening titles and abstracts (level 1 screening), 82 papers were selected for eligibility. After reading the full text (level 2 screening), 26 papers involving 1888 patients were included in the analysis, of which 10 evaluated multiple drugs (*Figure 1*). Specifically, 15 papers analysed the haemodynamic effect of dobutamine, 11 focused on levosimendan and 10 on milrinone. Overall, 728 patients were included in the analysis on dobutamine, 540 on levosimendan and 620 on milrinone (see Supplementary Table S2 for the number of patients for specific parameters).

### Study and patient characteristics

A summary of the study characteristics is reported in *Table 2*. Eleven publications (907 patients) were from the United States of America, 3 from Canada (397 patients), 1 from China (60 patients) and 21 (524 patients) from Europe. Twenty-six cohorts included acute HF patients, and 9 evaluated patients with low-output chronic HF. Twelve papers were retrospective analyses and 14 were prospective studies. The average age of patients ranged from 54 to 75 years old.

**Table 2:** Main characteristics of the selected studies

First author	Year	No. of patients	Drug	Dose (µg/kg/(min))	Timing	Country	Type of study	Acute/Chronic
Abramov^12^	2017	69	Milrinone	0.375 infusion	—	USA	Observ	Chronic
Baruch^13^	2001	19	Milrinone	50 bolus	24 h	USA	Trial	Acute
Bergh^14^	2010	29	Levosimendan	12 (bolus) and 0.2 mcg infusion	48 h	Europe	Trial	Acute
Bergh^14^	2010	31	Dobutamine	5 (bolus) and 10 infusion	48 h	Europe	Trial	Acute
Charisopoulpu^15^	2014	22	Milrinone	50 bolus	—	UK	Observ	Chronic
Follath^16^	2002	103	Levosimendan	12 (bolus) and 0.1 infusion	24 h	Europe	Trial	Acute
Follath^16^	2002	100	Dobutamine	5	24 h	Europe	Trial	Acute
Garcia-Gonzalez^17^	2006	11	Levosimendan	24 (bolus) and 0.1 infusion	—	Spain	Trial	Acute
Garcia-Gonzalez^17^	2006	11	Milrinone	5 (staring dose)	—	Spain	Trial	Acute
Guerrero-Orriach^18^	2020	30	Dobutamine	5 infusion	24 h	Spain	Observ	Acute
Guerrero-Orriach^18^	2020	30	Levosimendan	0.1 infusion	—	Spain	Observ	Acute
Karlsberg^19^	1996	30	Milrinone	50 (bolus) and 0.5 infusion	24 h	USA	Trial	Acute
Karlsberg^19^	1996	14	Dobutamine	2.5 uptitrated to 15 infusion	24 h	USA	Trial	Acute
Kieback^20^	1998	40	Dobutamine	—	3 h	Germany	Trial	Chronic
Lewis^21^	2018	50	Milrinone	—	3 days	USA	Observ	Acute
Lewis^21^	2018	50	Dobutamine	—	—	USA	Observ	Acute
Lilleberg^22^	2006	11	Levosimendan	0.1 infusion	24 h	Finland	Trial	Chronic
Loh^23^	2001	60	Milrinone	50 (bolus) and 0.5 (infusion)	—	USA	Observ	Acute
Mathew^24^	2001	96	Dobutamine	—	24 h	Canada	Trial	Acute
Mathew^24^	2021	96	Milrinone	—	24 h	Canada	Trial	Acute
Metra^25^	2002	29	Dobutamine	—	—	Italy	Trial	Acute
Moertl^26^	2005	38	Levosimendan	0.1 infusion	24 h	Austria	Trial	Acute
Mokhtari^27^	2008	10	Dobutamine	6 infusion	24 h	Germany	Observ	Acute
Niemien^28^	2000	95	Levosimendan	6–24 (bolus) and 0.05-0.2 infusion	24 h	Europe	Trial	Chronic
Niemien^28^	2000	20	Dobutamine	5 infusion	24 h	Europe	Trial	Chronic
Nunez^29^	1998	9	Milrinone	0.5 infusion	24 h	USA	Trial	Acute
Rodenas-Alesina^30^	2023	205	Milrinone	—	—	Canada	Observ	Acute
Russ^31^	2007	56	Levosimendan	0.1 infusion	48 h	Germany	Observ	Acute
Slawsky^32^	2000	98	Levosimendan	6 (bolus) and 0.1 infusion	6 h	USA	Trial	Chronic
Sun^33^	2023	60	Levosimendan	12.5 mg total infusion	7 days	China	Observ	Acute
Tokuda^34^	2005	9	Levosimendan	12 (bolus) and 0.2 infusion	24 h	Australia	Observ	Acute
Velez-Roa^35^	2003	9	Dobutamine	10 infusion	48 h	Belgium	Trial	Chronic
Wimmer^36^	1999	10	Dobutamine	4.5 infusion	—	Austria	Trial	Chronic
Yamani^37^	2001	269	Dobutamine	—	—	USA	Observ	Acute
Yamani^37^	2001	60	Levosimendan	—	—	USA	Observ	Acute

### Quality assessment results

According to the NIH-QAT evaluation of the studies included in the final analysis, 11 had a low risk of bias, 14 had a moderate risk of bias and 1 had a high risk of bias. The items less frequently met were item 3, item 5 and item 8, while the most commonly met were item 1 and item 10. A comprehensive assessment of the risk of bias is reported in *Table 3*. Evidence certainty for all primary haemodynamic outcomes was assessed using the GRADE approach (Supplementary Table S3).

**Table 3:** Quality assessment of the studies included using the NIH-QAT

First author	Publication year	Q1	Q2	Q3	Q4	Q5	Q6	Q7	Q8	Q9	Q10	Q11	Q12	Total quality score	Risk of bias
Abramov^12^	2017	✓	✓	✓	✓	✗	✗	✓	✗	✗	✓	✗	✓	7	Moderate
Baruch^13^	2001	✓	✓	✗	✓	✗	✓	✓	✓	✓	✓	✓	✓	10	Low
Bergh^14^	2010	✓	✓	✗	✓	✗	✓	✓	✓	✓	✓	✗	✓	9	Low
Charisopoulpu^15^	2014	✓	✗	✓	✓	✗	✓	✓	✗	✓	✓	✗	✓	8	Moderate
Follath^16^	2002	✓	✓	✗	✓	✓	✓	✓	✗	✗	✓	✗	✓	8	Moderate
Garcia-Gonzalez^17^	2006	✓	✓	✓	✓	✗	✓	✓	✗	✓	✓	✓	✓	10	Low
Guerrero-Orriach^18^	2020	✓	✓	✓	✓	✗	✓	✓	✓	✓	✓	✗	✓	10	Low
Karlsberg^19^	1996	✓	✓	✓	✓	✗	✗	✓	✗	✓	✓	✗	✓	8	Moderate
Kieback^20^	1998	✓	✗	✗	✓	✗	✓	✓	✗	✓	✓	✗	✓	7	Moderate
Lewis^21^	2018	✓	✓	✓	✗	✗	✓	✗	✗	✓	✓	✗	✓	7	Moderate
Lilleberg^22^	2006	✓	✓	✗	✓	✗	✓	✓	✓	✓	✓	✗	✓	9	Low
Loh^23^	2001	✓	✓	✗	✓	✓	✓	✓	✗	✓	✓	✗	✓	9	Low
Mathew^24^	2021	✓	✓	✓	✓	✓	✓	✓	✓	✓	✓	✓	✓	12	Low
Metra^25^	2002	✓	✓	✗	✓	✗	✓	✓	✗	✓	✓	✓	✓	9	Low
Moertl^26^	2005	✓	✓	✗	✓	✗	✓	✓	✗	✓	✓	✗	✓	8	Moderate
Mokhtari^27^	2008	✓	✓	✗	✗	✗	✗	✗	✗	✓	✓	✗	✓	5	High
Niemen^28^	2000	✓	✓	✓	✗	✓	✗	✓	✓	✓	✓	✓	✗	9	Low
Nunez^29^	1998	✓	✗	✗	✓	✗	✓	✓	✗	✓	✓	✓	✓	8	Moderate
Rodenas-Alesina^30^	2023	✓	✓	✓	✓	✓	✗	✗	✗	✓	✓	✗	✓	8	Moderate
Russ^31^	2007	✓	✓	✗	✓	✗	✗	✗	✗	✓	✓	✗	✓	6	Moderate
Slawsky^32^	2000	✓	✓	✗	✓	✗	✓	✓	✓	✓	✓	✓	✓	10	Low
Sun^33^	2023	✓	✓	✓	✓	✓	✗	✗	✗	✓	✓	✗	✓	8	Moderate
Tokuda^34^	2005	✓	✓	✓	✓	✓	✓	✓	✓	✗	✓	✗	✓	10	Low
Velez-Roa^35^	2003	✓	✗	✗	✗	✗	✓	✓	✗	✓	✓	✓	✓	7	Moderate
Wimmer^36^	1999	✓	✓	✗	✓	✗	✓	✓	✗	✓	✓	✓	✓	9	Moderate
Yamani^37^	2001	✓	✓	✗	✓	✓	✓	✗	✗	✓	✓	✗	✓	8	Moderate

### Outcomes results

**Figure 2:**
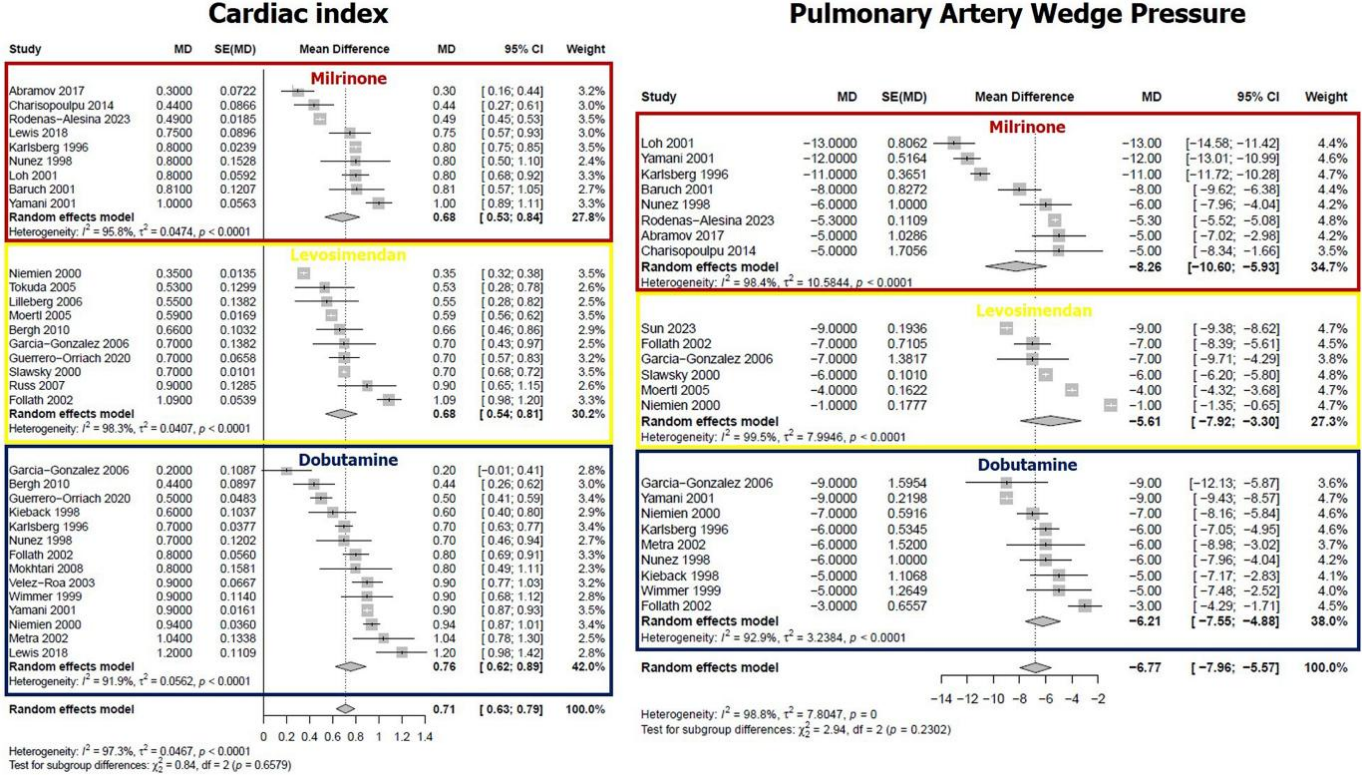
Effects of milrinone, levosimendan, and dobutamine on cardiac index (left) and pulmonary artery wedge pressure (right). Individual and pooled mean differences shown with 95% confidence interval. Heterogeneity and *P*-values for each drug are reported below each panel. Overall heterogeneity and between-drug comparison *P* value in the bottom row.

**Figure 3:**
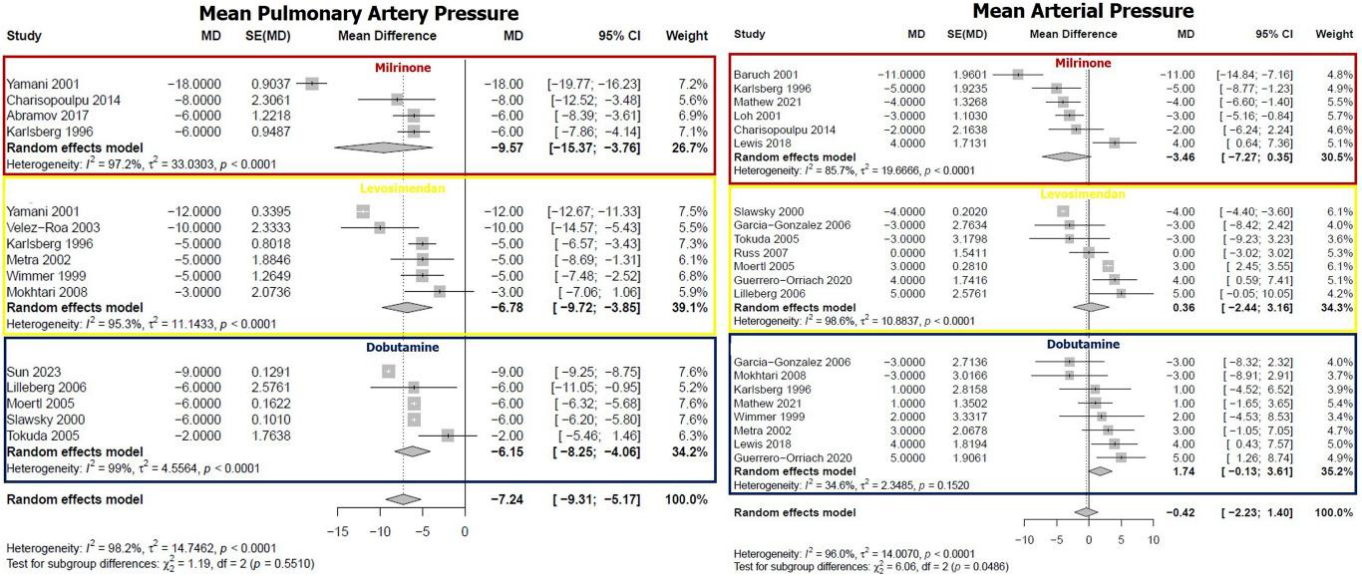
Effects of milrinone, levosimendan, and dobutamine on mean pulmonary artery pressure (left) and mean arterial pressure (right). Individual and pooled mean differences shown with 95% confidence interval. Heterogeneity and *P*-values for each drug are reported below each panel. Overall heterogeneity and between-drug comparison *P* value in the bottom row.

**Figure 4:**
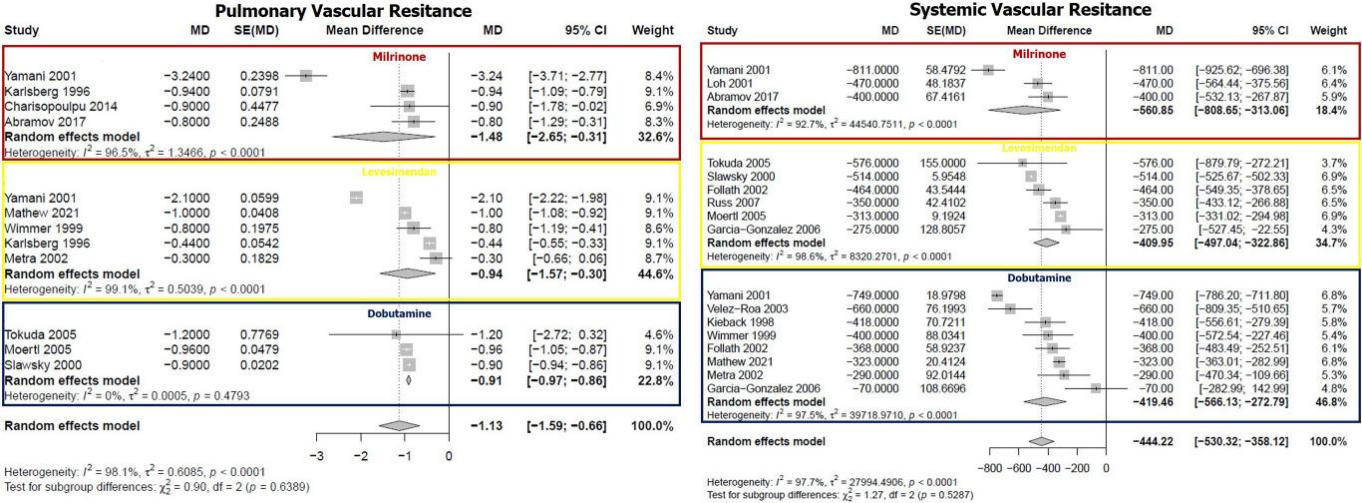
Effects of milrinone, levosimendan, and dobutamine on pulmonary vascular resistance (left) and systemic vascular resistance (right). Individual and pooled mean differences shown with 95% confidence interval. Heterogeneity and *P*-values for each drug are reported below each panel. Overall heterogeneity and between-drug comparison *P* value in the bottom row.

Overall, all the study drugs showed a significant improvement in CI. Specifically, the magnitude of increase in CI in HF patients treated with dobutamine was +0.76 (+0.62 to +0.89) L/min/m^2^, the increase in those treated with levosimendan was +0.68 (+0.54 to +0.81) L/min/m^2^, and for patients treated with milrinone the improvement was +0.68 (+0.53 to +0.84) L/min/m^2^. Regarding PAWP, dobutamine reduced PAWP by an average of −6.21 (−7.96 to −4.88) mmHg, levosimendan by −5.61 (−7.92 to −3.3) while the reduction in patients treated with milrinone was −8.26 (−10.60 to −5.93) (*Figure 2*). All the study drugs reduced mPAP. In particular, the reductions with dobutamine, levosimendan, and milrinone were −6.15 (−8.25 to −4.06) mmHg, −6.78 (−9.72 to −3.85) mmHg, and −9.57 (−15.37 to −3.76) mmHg, respectively. Significant differences were observed in the effect on MAP according to the drug administered. Dobutamine and levosimendan caused a non-significant increase in MAP [+1.74 (−0.13 to +3.61) and +0.36 (−2.44 to +3.16) mmHg, respectively], while milrinone showed a trend towards a reduction in MAP [−3.46 (−7.27 to +0.35)] (*Figure 3*). Concerning PVR, milrinone led to a significant reduction in the values [−1.48 (−2.65 to −0.31)]. Interestingly, the administration of both dobutamine and levosimendan determined a significant reduction in PVR [−0.91 (−0.97 to −0.86) and −0.94 (−1.57 to −0.30) Woods units, respectively] as well. Finally, with all the agents investigated, a significant reduction in SVR was observed (*Figure 4*). There was no difference amongst the different drugs in terms of improvement in CI (*P* = .658), PAWP (*P* = .230), mPAP (*P* = .551), PVR (*P* = .639), and SVR (*P* = .529). The only haemodynamic parameter that changed significantly according to the drug tested was MAP (*P* = .0486). Overall, the heterogeneity was high for all the variables studied (*I^2^* from 96% to 98.8%, *P* < .001). Still, for a few specific analyses, this was low (effect of dobutamine on MAP and SVR: *I^2^* 34.6%, *P* = .152 and *I^2^* 0%, *P* = .479, respectively). A summary of the main results is reported in the *Graphical Abstract*. For the primary endpoint (change in CI), none of the tested moderators significantly explained the between-study variability (all *P* ≥ .13; *R*^2^ = 0–20%), and residual heterogeneity remained high (*I*^2^ ≈ 95%) (Supplementary Table S4). Influence diagnostics and leave-one-out analyses did not identify any influential studies, with Cook’s distances consistently below conventional thresholds and minimal variation in the pooled CI increase across models (+0.70 to +0.73 L/min/m^2^ vs +0.71 L/min/m^2^ in the main analysis) (Supplementary Table S5). For MAP, mPAP, PAWP, and PVR, baseline values significantly predicted the magnitude of change (*P* ≈ .0002, .0002, .0007, and <.0001, respectively), explaining a relevant portion of heterogeneity (R^2^ ≈ 34–79%), although substantial residual heterogeneity persisted (*I*^2^ ≥ 78%). Baseline SVR was not significantly associated with changes in SVR (*P* = .13; Supplementary Table S6).

### Sensitivity analysis

Sensitivity analyses, separately examining the potentially different effects of dobutamine, levosimendan and milrinone in patients with acute HF and chronic low-output HF, showed that CI improved more in the chronic setting when treated with dobutamine compared with the other studied drugs. Moreover, a lower heterogeneity was observed in the studies focusing on chronic low-output patients was observed (Supplementary Figures). When the analysis was restricted to studies with clearly reported dosing regimens, the pooled haemodynamic effects remained highly consistent with the main results. For CI, the pooled MD changed minimally from +0.71 L/min/m^2^ (95% CI 0.63–0.79) in the full dataset to +0.68 L/min/m^2^ (0.59–0.76) in the restricted dataset.

Similar concordance was observed for MAP (−0.42 [−2.23; 1.40] vs −1.07 [−3.24; 1.10]), mPAP (−7.24 [−9.31; −5.17] vs −6.09 [−7.20; −4.98]), PAWP (−6.77 [−7.96; −5.57] vs −6.58 [−8.00; −5.15]) and PVR (−1.13 [−1.59; −0.66] vs −0.82 [−0.99; −0.64]).

In all cases, confidence intervals overlapped widely and the direction of effect remained unchanged, confirming the robustness of the findings despite heterogeneous dosing information. When the analysis was stratified by study design (randomized controlled trials vs observational studies), pooled haemodynamic responses remained highly consistent (Supplementary Table S8). For CI, the pooled MD was +0.71 L/min/m^2^ (95% CI 0.57–0.85; *k* = 13) in observational studies and +0.71 L/min/m^2^ (95% CI 0.62–0.81; *k* = 20) in randomized trials (*P* for subgroup differences = 0.98). For mPAP, the pooled MD was −8.47 mmHg (95% CI −12.58 to −4.35; *k* = 7) in observational studies and −5.99 mmHg (95% CI −6.15 to −5.82; *k* = 8) in randomized trials (*P* = .24). Observational cohorts showed numerically larger reductions in PAWP, PVR and SVR (e.g. PVR −1.71 [95% CI −2.66 to −0.75] vs −0.79 Wood units [95% CI −0.98 to −0.59] in RCTs; *P* = .06), but confidence intervals overlapped widely and the direction of effect remained unchanged across all endpoints (Supplementary Table S8).

### Assessment of publication bias

The six funnel plots did not show relevant asymmetry, and Egger’s regression tests revealed no statistically significant small-study effects (*P*-values ranging from .32 to .84; Supplementary Figure S7 and Supplementary Table S9).

## Discussion

To our knowledge, this is the first meta-analysis to evaluate the haemodynamic effects of the most used inotropic drugs in patients with HF, providing an estimation of the magnitude of improvement achieved with dobutamine, levosimendan and milrinone. While the significant increase in CI with all three agents was expected, surprisingly, no significant differences were observed amongst the drugs in terms of CI augmentation. Furthermore, all drugs significantly reduced the PAWP, thereby improving pulmonary circulation, with associated reductions in both mPAP and PVR. Interestingly, significant differences emerged regarding their effects on MAP: levosimendan and, especially, dobutamine showed a trend towards MAP elevation, whereas milrinone slightly reduced MAP. Finally, SVR was consistently decreased following administration of each agent.

The main goal of inotropic support is to provide a clinical benefit from the established positive effect on CI. In this meta-analysis, we quantified with precision the degree of haemodynamic improvement achievable. Compared with other commonly used interventions, such as intra-aortic balloon pump (IABP), the CI increase obtained with inotropes was markedly superior. Indeed, observational studies have shown that the CI increment with IABP ranges from 0.26 L/min/m^2^ to 0.6 L/min/m^2^, depending on the baseline haemodynamic condition.^38,39^ This is in line with current indications to initiate inotropic therapy before mechanical circulatory support in patients with decompensated HF.^40^

A noteworthy finding was the PAWP reduction observed even with dobutamine, an inotrope without a prominent vasodilator effect especially at doses ≥5 mcg/Kg/min and frequently used in the treatment of low cardiac output. In HF with reduced ejection fraction, pulmonary congestion is primarily driven by high PAWP, secondary to impaired LV emptying and, possibly, mitral regurgitation, rather than impaired relaxation. Dobutamine enhances cardiac LV contractility, improving emptying and, consequently, reducing LV end-diastolic pressure even in the absence of significant vasodilation on systemic or pulmonary arteries.^41^ As a positive consequence, pulmonary circulation improves, with a reduction in PVR and mPAP, facilitating decongestion. These findings support the clinical use of dobutamine and inotrope therapy overall in patients with diuretic-resistant congestion, to enhance renal function and diuresis.^42^ Despite the very high heterogeneity, additional random-effects meta-regression and influence analyses showed that no single study drove the observed haemodynamic effects, and pooled estimates were essentially unchanged in leave-one-out analyses. Baseline haemodynamic values partially explained the variability in the magnitude of change for MAP, mPAP, PAWP, and PVR, but residual heterogeneity remained substantial, likely reflecting unmeasured clinical and methodological differences across studies. This was anticipated and reflects the variability in how these therapies are used in clinical practice. Indeed, in a global survey involving 60 countries, wide disparities in the treatment of cardiogenic shock were recorded. For example, even for dobutamine, the most widely used inotrope, there was great variability in its use as a first-line therapy, with prescription rates ranging from as low as 13% to as high as 88% across centres. Dosage regimens also varied substantially. For noradrenaline, the maximum dose was ≤0.15 µg/kg/min for 29% of respondents, 0.15–0.5 µg/kg/min for 53%, and >15 µg/kg/min for 18%.^43^ Similarly, the parameters used to guide changes in inotropic therapy significantly vary in routine clinical practice. In a real-world survey involving 839 physicians, only 50% cited haemodynamic variables as their primary guide for reducing inotropic support, while others prioritized clinical status and side effects of inotropes.^44^ Patient heterogeneity in acute HF is another key contributor to the variability in response to inotropes. The clinical scenario—whether triggered by an acute event (e.g. myocardial infarction, myocarditis, arrhythmias) or by progressive decompensation of chronic HF—may significantly influence the haemodynamic response to inotropes.^45^ The underlying aetiology of LV dysfunction (ischaemic vs non-ischaemic) is also relevant. In a post-hoc analysis of a randomized clinical trial, milrinone was associated with worse outcomes in ischaemic HF, while its effect in non-ischaemic patients was neutral.^46^ This heterogeneity of the acute HF setting is also reflected in our findings, as sensitivity analyses restricted to chronic low-output HF populations showed significantly lower heterogeneity. Future focused, high quality, prospective clinical trials should stratify patients according to phenotype to better assess haemodynamic responses.

The discordance between MAP and CI changes with inotrope use is clinically meaningful. Indeed, while CI invariably improved with all agents, MAP slightly declined with milrinone. This result underscores that MAP is not a reliable surrogate of tissue perfusion, which depends more directly on CI. Prior observational studies, indeed, have demonstrated that MAP is not accurate in identifying hypoperfusion, assessed via lactate levels, and is not strongly correlated with CI.^47,48^

In the sensitivity analysis, we focused on chronic low-output HF patients, as they may require inotropes to test the reversibility of pulmonary hypertension while on the heart transplant waiting list, to prevent hospitalizations for HF, and to improve quality of life. We observed that dobutamine demonstrated greater efficacy than levosimendan in patients with chronic HF. Patients with long-standing HF are more likely to have impaired intracellular signalling, potentially diminishing the response to calcium sensitizers or phosphodiesterase inhibitors, which require intact transduction pathways to exert their effects.^49^ Although inotrope-induced haemodynamic responses are dose-dependent at a pharmacological level, restricting the analysis to studies with clearly reported dosing showed that pooled effects were virtually unchanged across all endpoints. These results indicate that dosing inconsistencies did not materially influence the overall conclusions, likely reflecting the relatively narrow dosing ranges used in clinical practice and the acute nature of most haemodynamic assessments. Although the body of evidence combines randomized trials and observational cohorts, stratified analyses did not show any statistically significant modification of treatment effects by study design under a random-effects model. Haemodynamic responses were directionally concordant and of similar magnitude in RCTs and observational studies across all endpoints, suggesting that pooling these designs is unlikely to have biased the overall estimates.

In the light of the broadly comparable effect of these drugs in terms of CI increase, LV unloading and pulmonary circulation improvement, the inotrope choice in specific clinical scenarios might be guided by other factors, such as pharmacokinetics. For instance, levosimendan may be preferred for patients who would benefit from a long-acting effect without constant infusion, especially when the main aim is a reduction of the risk of HF hospitalization.^50^

The apparent discordance between the positive haemodynamic effect of inotropes and the lack of beneficial effects on strong clinical outcomes (e.g. mortality) remains a matter of debate. Multiple reasons might contribute to this discrepancy, including adverse effects of inotropes, inappropriate trial design, significant variability in patient characteristics and treatment protocols. Among the adverse effects of inotropes, it has been established that both heart rate and myocardial contractility are key determinants of myocardial oxygen consumption, contributing approximately 50–70% and 15–25%, respectively.^51^ Moreover, the risk of arrhythmic events in patients treated with inotropes is not negligible. A post-hoc analysis of the DOREMI trial showed that up to 47.9% of patients experienced clinically significant arrhythmic events—either supraventricular or ventricular—when treated with dobutamine or milrinone, with no significant difference between the two agents.^52^ Future clinical research should focus on improved phenotyping to identify patients most likely to benefit from active treatment, use run-in phases to exclude patients with low tolerance to these agents and incorporate biomarkers and advanced imaging, alongside invasive haemodynamic assessment, to better evaluate therapeutic efficacy.^53^

### Limitations

This study has several limitations. The high heterogeneity of the available studies limited the robustness of the results, mirroring variability both in patient characteristics and in how inotropes were administered across different settings. Detailed analyses on HF aetiology, comorbidities and concomitant beta-blocker therapy were not feasible because only a minority of the included studies provided these data. Although we were able to perform sensitivity analyses restricted to studies with clearly reported dosing regimens, a formal dose–response analysis or stratification by dose level and infusion duration could not be conducted, as dosing schemes were highly heterogeneous and many studies did not provide sufficiently granular information on inotrope titration. Additionally, studies investigating other drugs (e.g. adrenaline, dopamine, enoximone) were not numerous enough to allow meaningful comparisons. The concomitant use of other haemodynamically active drugs (vasopressor, diuretics) could not systematically be ruled out, as such treatments are often necessary in clinical practice. Lastly, although invasive haemodynamic is currently considered the gold standard, measurements may vary across centres, potentially affecting consistency.

## Conclusions

Inotropic support with dobutamine, levosimendan and milrinone consistently led to improvements in invasively assessed haemodynamic parameters in patients with low-output HF. No clear superiority emerged amongst the three drugs in terms of the magnitude of haemodynamic benefit, even if in the presence of high heterogeneity, except for differences in MAP and for response to dobutamine and levosimendan in patients with chronic HF. Randomized clinical trials enrolling carefully phenotyped patients are warranted to define the specific haemodynamic response to each agent and define their prognostic effect.

## Supplementary Material

xvag120_Supplementary_Data

## Declarations

### Data availability

No data were generated or analysed for this manuscript.

### Pre-registered clinical trial number

None supplied.
