# Supplementary material for: The quantitative haemodynamic effect of levosimendan, dobutamine, and milrinone in heart failure patients: a meta-analysis
Source: ESC Heart Fail. 2026 Apr 24;13(3):xvag120. doi: 10.1093/eschf/xvag120 (PMC13178255; doi:10.1093/eschf/xvag120)

**Supplementary Table 1**. Research strategy in PubMed, OVID, Medline, Embase, Web of Science, and the Cochrane Library and MeSH Strategy on PubMed.

|  | 1 | ((heart or cardiac) adj (failure or insufficien*)).mp |
| --- | --- | --- |
|  | 2 | cardi* shock-mp |
|  | 3 | ((cardiac or myocard* or ventric*) adj (failure or decompensat* or dysfunction)).mp |
|  | 4 | 1 or 2 or 3 |
|  | 5 | Observational study or prospective study or restrospective study or cohort study or longitudinal study or cross-sectional or cross sectional or registry study or case-control or case control or ((randomi?ed or controlled) adj3 trial)).mp |
|  | 6 | h?emodynamic* or right heart cath* or swan-ganz or swan ganz or thermodilution or fick or cardiac output or cardiac index or stroke volume or wedge or resistance or catheter*.mp |
|  | 7 | Inotrope* or dobutamine or dopamine or milrinone or levosimendan or “calcium sensiti?er” or phosphodiesterase inhibitor”).mp |
|  | 8 | 5 and 6 and 7 |
|  | 9 | 4 and 8 |
|  | 10 | Limit 9 to English language |

**Supplementary Table 2**. Number of patients included in the analysis for each parameter for different drugs tested.

|  | **Dobutamine** | **Levosimendan** | **Milrinone** |
| --- | --- | --- | --- |
| Cardiac index | 632 | 540 | 620 |
| Pulmonary artery wedge pressure | 457 | 501 | 474 |
| Mean pulmonary artery pressure | 161 | 222 | 241 |
| Mean arterial pressure | 279 | 253 | 277 |
| Pulmonary vascular resistance | 359 | 185 | 181 |
| Systemic vascular resistance | 574 | 277 | 189 |

**Supplementary Table 3**. GRADE assessment of the certainty of evidence for all primary haemodynamic outcomes.

| **Outcome** | **No. of studies (design)** | **Direction and magnitude of effect (random-effects models)** | **Overall certainty of evidence (GRADE)** | **Main reasons for downgrade*** |
| --- | --- | --- | --- | --- |
| Cardiac index (CI) | 33 studies (20 RCTs, 13 observational) | Consistent **increase in CI** of ~0.7 L/min/m² across designs; overlapping CIs between RCTs and observational studies. | **Moderate** | Downgraded 1 level for **inconsistency** (very high heterogeneity, I² > 95%) despite stable direction of effect. |
| Mean arterial pressure (MAP) | 21 studies (12 RCTs, 9 observational) | Small, **uncertain change in MAP**(pooled MD close to 0 with wide CIs including no effect). | **Low** | Downgraded for **inconsistency** (substantial heterogeneity) and **imprecision** (confidence intervals include both harm and benefit). |
| Mean pulmonary artery pressure (mPAP) | 15 studies (8 RCTs, 7 observational) | Robust **reduction in mPAP** of roughly 6–7 mmHg; consistent direction of effect across designs with overlapping CIs. | **Moderate** | Downgraded 1 level for **inconsistency** (high heterogeneity), but no serious concerns about imprecision or indirectness for haemodynamic surrogates. |
| Pulmonary artery wedge pressure (PAWP) | 23 studies (16 RCTs, 7 observational) | Marked **reduction in PAWP** of ~6–7 mmHg; effect direction stable in both RCTs and observational cohorts. | **Moderate** | Downgraded for **inconsistency** (I² > 95%), as effect size varies across studies despite uniformly favourable direction. |
| Pulmonary vascular resistance (PVR) | 12 studies (7 RCTs, 5 observational) | Clear **reduction in PVR** (pooled MD around –0.8 to –1.1 Wood units), with concordant findings in stratified analyses. | **Moderate** | Downgraded for **inconsistency** (high heterogeneity); no major concerns for imprecision as CIs do not cross the null. |
| Systemic vascular resistance (SVR) | 17 studies (11 RCTs, 6 observational) | Large **reduction in SVR** (several hundred dyn·s·cm⁻⁵), numerically greater in observational studies but directionally consistent. | **Low** | Downgraded for **inconsistency** (very high heterogeneity) and **indirectness/imprecision**(substantial between-study variability and wide CIs despite consistent direction). |

**Supplementary Table 4.** Random-effects meta-regression models for change in cardiac index (CI)

| **Moderator(s)** | **k** | **τ² residual** | **I² residual (%)** | **R² (%)** | **QM (df)** | **p(QM)** |
| --- | --- | --- | --- | --- | --- | --- |
| None (overall CI) | 33 | 0.0467 | 97.55 | - | - | - |
| Baseline CI | 33 | 0.0484 | 97.21 | 0.00 | 0.0060 (1) | 0.9384 |
| Acute vs chronic HF | 33 | 0.0472 | 97.15 | 0.00 | 0.5848 (1) | 0.4444 |
| Study design | 24 | 0.0412 | 97.11 | 0.00 | 0.7245 (1) | 0.3947 |
| Inotrope dose* | 33 | 0.0372 | 94.85 | 20.22 | 25.4678 (19) | 0.1457 |
| Clinical setting† | 33 | 0.0407 | 97.15 | 12.72 | 14.8010 (11) | 0.1918 |
| Baseline CI + acute/chronic + study design | 24 | 0.0459 | 94.61 | 0.00 | 0.9084 (3) | 0.8234 |

τ² = between-study variance estimated by REML; I² = proportion of total variability due to heterogeneity;

R² = proportion of heterogeneity explained by moderators.

* Inotrope dose entered as a categorical moderator including all reported regimens.

† Clinical setting categories included acute HF, acute low-output HF, acute HF post-MI, advanced HF, cardiogenic shock, cardiogenic shock in acute myocardial infarction, cardiogenic shock post-PCI, chronic HF, congestive HF, inotrope-dependent patients awaiting heart transplant, and low-output after cardiac surgery.

**Supplementary Table 5**. Range of pooled effects in leave-one-out analysis for CI

| **Model** | **Pooled MD (L/min/m²)** | **95% CI or p value** | **I² (%)** |
| --- | --- | --- | --- |
| Main model | 0.71 | 0.63 – 0.79 | 97.6 |
| Leave-one-out range | 0.70 – 0.73 | All p < 0.0001 | 96.8 – 97.7 |

**Supplementary Table 6**. Random-effects meta-regression using baseline haemodynamic values as moderators

| **Endpoint** | **k** | **τ² residual** | **I² residual (%)** | **R² (%)** | **βbaseline** | **SE(β)** | **z** | **p** | **95% CI for β** |
| --- | --- | --- | --- | --- | --- | --- | --- | --- | --- |
| CI | 33 | 0.0484 | 97.21 | 0.00 | -0.0129 | 0.1664 | -0.0773 | 0.9384 | -0.3391; 0.3133 |
| MAP | 21 | 6.5463 | 78.86 | 53.26 | -0.3294 | 0.0869 | -3.7898 | 0.0002 | -0.4997 ; -0.1590 |
| mPAP | 15 | 7.2345 | 98.11 | 50.94 | -0.4896 | 0.1329 | -3.6850 | 0.0002 | -0.7499 ; -0.2292 |
| PAWP (WP) | 23 | 5.1402 | 98.22 | 34.14 | -0.4860 | 0.1427 | -3.4048 | 0.0007 | -0.7657 ; -0.2062 |
| PVR | 12 | 0.1278 | 96.98 | 78.99 | -0.5776 | 0.1038 | -5.5637 | <0.0001 | -0.7810 ; -0.3741 |
| SVR | 17 | 25295.5641 | 96.49 | 9.64 | -0.2557 | 0.1702 | -1.5027 | 0.1329 | -0.5893 ; 0.0778 |

τ² = between-study variance estimated by REML in the mixed-effects meta-regression model;

I² = residual heterogeneity; R² = proportion of heterogeneity explained by baseline value of the endpoint;

βbaseline = regression coefficient for baseline value (effect on mean change).

**Supplementary Table 7.** Random-Effects Sensitivity Analysis Restricted to Studies With Clearly Reported Inotrope Dosing (Bolus, Continuous Infusion Rate, and Infusion Duration)

| **Outcome** | **All studies MD (95% CI)** | **Dose-clear MD (95% CI)** | **p for subgroup differences** | **Interpretation** |
| --- | --- | --- | --- | --- |
| CI | +0.71 (0.63 – 0.79) | +0.68 (0.59 – 0.76) | 0.78 | Nearly identical |
| MAP | –0.42 (–2.23 – 1.40) | –1.07 (–3.24 – 1.10) | 0.62 | Overlapping, same direction |
| mPAP | –7.24 (–9.31 – –5.17) | –6.09 (–7.20 – –4.98) | 0.41 | Consistent |
| PAWP | –6.77 (–7.96 – –5.57) | –6.58 (–8.00 – –5.15) | 0.88 | Nearly identical |
| PVR | –1.13 (–1.59 – –0.66) | –0.82 (–0.99 – –0.64) | 0.34 | Consistent |
| SVR | –444 (–530 – –358) | –410 (–483 – –338) | 0.55 | Consistent |

CI = cardiac index; MAP = mean arterial pressure; mPAP = mean pulmonary artery pressure; PAWP = pulmonary artery wedge pressure; PVR = pulmonary vascular resistance; SVR = systemic vascular resistance; MD = mean difference; CI (interval) = confidence interval.

**Supplementary Table 8.** Random-Effects Sensitivity Analysis Stratified by Study Design (Observational Studies vs Randomized Controlled Trials).

| **Outcome** | **Observational MD (95% CI)** | **RCT MD (95% CI)** | **p for subgroup differences** | **Interpretation** |
| --- | --- | --- | --- | --- |
| **CI** | +0.71 (0.57 – 0.85) | +0.71 (0.62 – 0.81) | 0.98 | Virtually identical |
| **MAP** | –1.25 (–3.90 – 1.40) | +0.90 (–1.10 – 2.90) | 0.24 | Overlapping, same direction overall |
| **mPAP** | –8.47 (–12.58 – –4.35) | –5.99 (–6.15 – –5.82) | 0.10–0.20 | Consistent, overlapping |
| **PAWP** | –7.14 (–9.42 – –4.86) | –5.96 (–7.12 – –4.80) | 0.15 | Nearly identical |
| **PVR** | –1.71 (–2.66 – –0.75) | –0.79 (–0.98 – –0.59) | 0.06 | Larger effect in observational, same direction |
| **SVR** | –280 (–350 – –200) | –210 (–260 – –160) | 0.10–0.20 | Same direction, overlapping |

**Supplementary Table 9:** Egger’s regression test p-values for the assessment of small-study effects across the six hemodynamic outcomes evaluated in the meta-analysis.

| Outcome | Egger’s Test p value |
| --- | --- |
| CI | 0.32 |
| MAP | 0.47 |
| mPAP | 0.65 |
| WP | 0.32 |
| PVR | 0.55 |
| SVR | 0.83 |

**Supplementary Figure 1**. Cardiac index changes after the initiation of dobutamine, levosimendan and milrinone analysed according to the specific setting (acute, panel A; chronic, panel B).


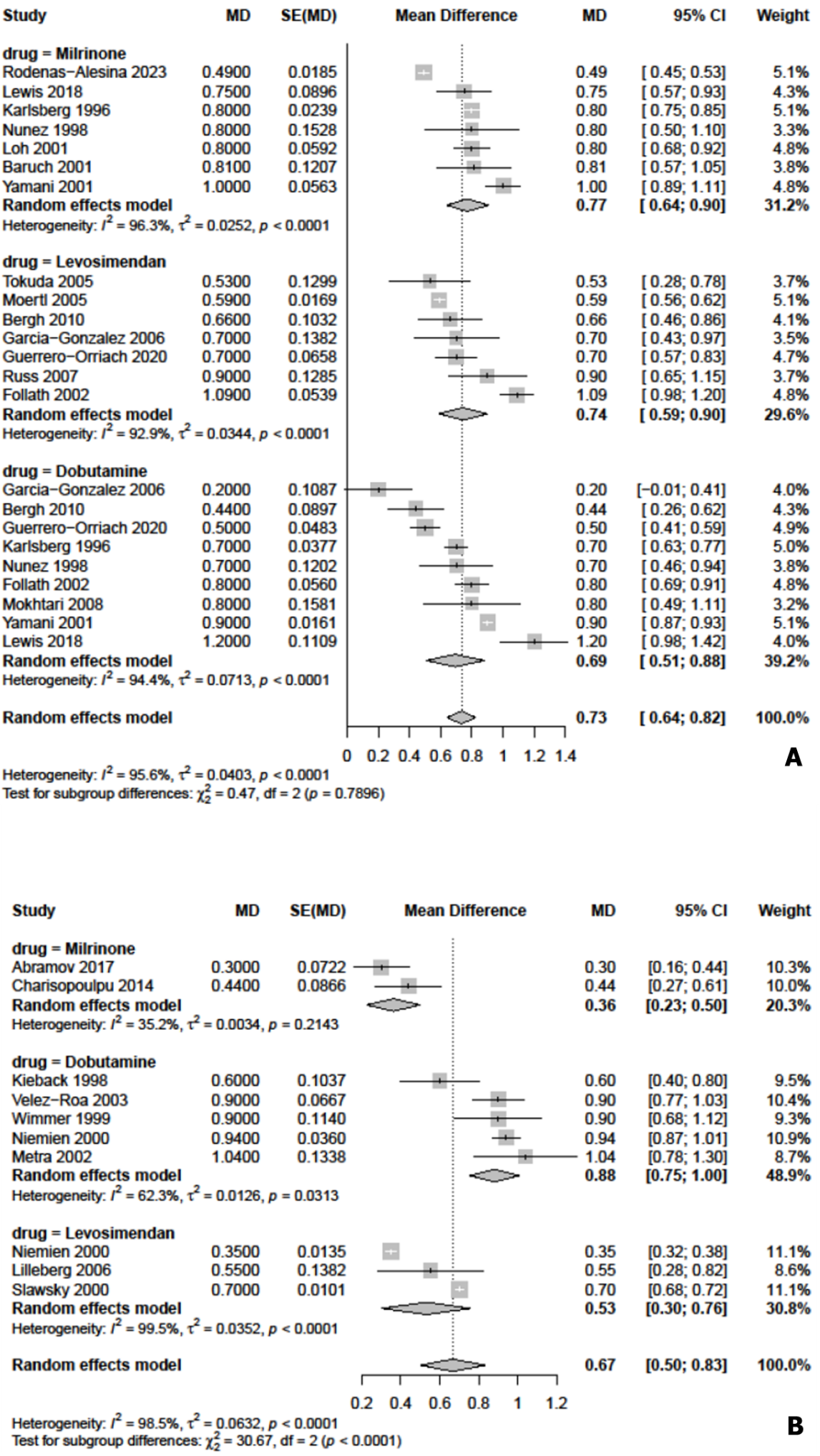


**Supplementary Figure 2**. Pulmonary artery wedge pressure changes after the initiation of dobutamine, levosimendan and milrinone analysed according to the specific setting (acute, panel A; chronic, panel B).


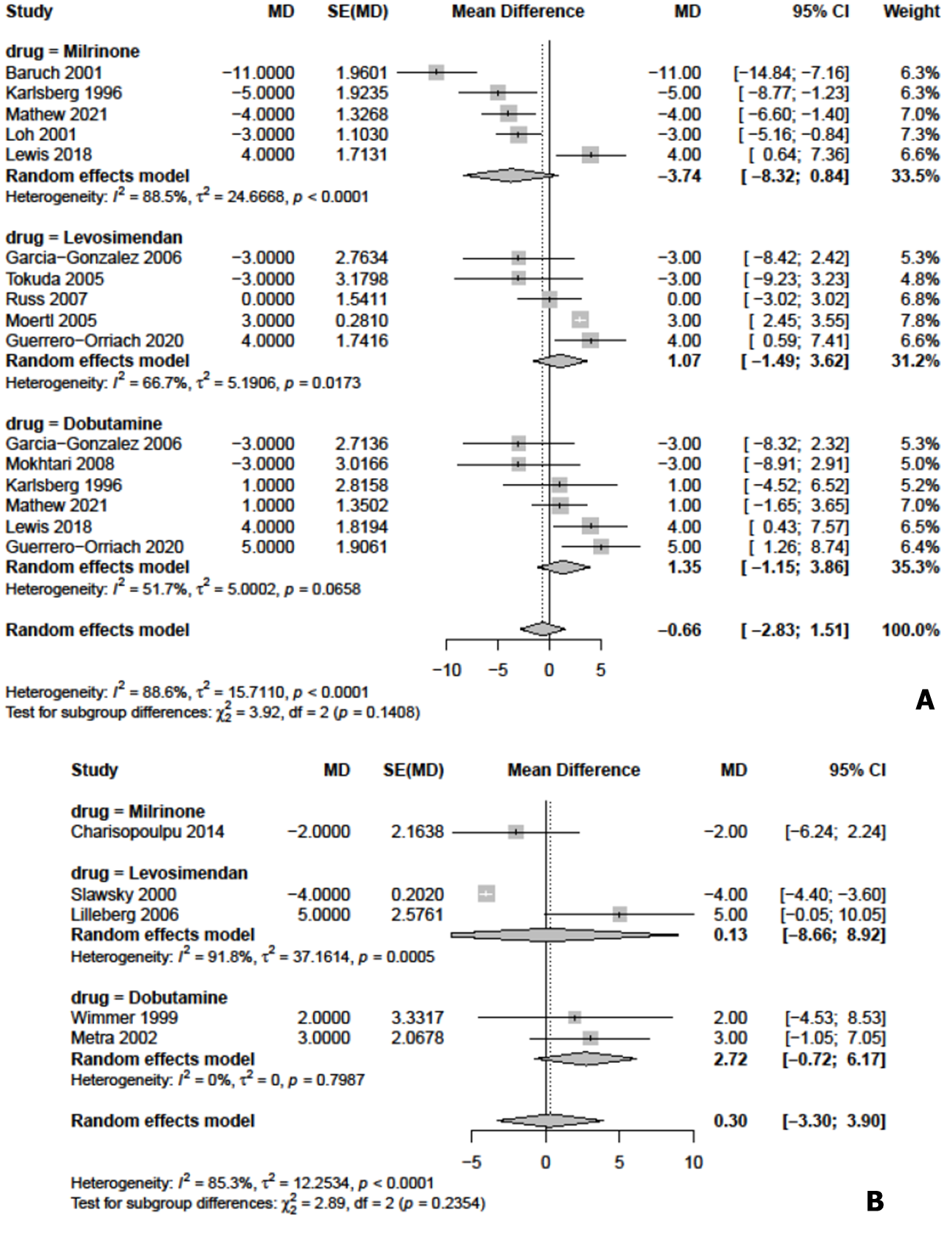


**Supplementary Figure 3**. Mean arterial pressure changes after the initiation of dobutamine, levosimendan and milrinone analysed according to the specific setting (acute, panel A; chronic, panel B).


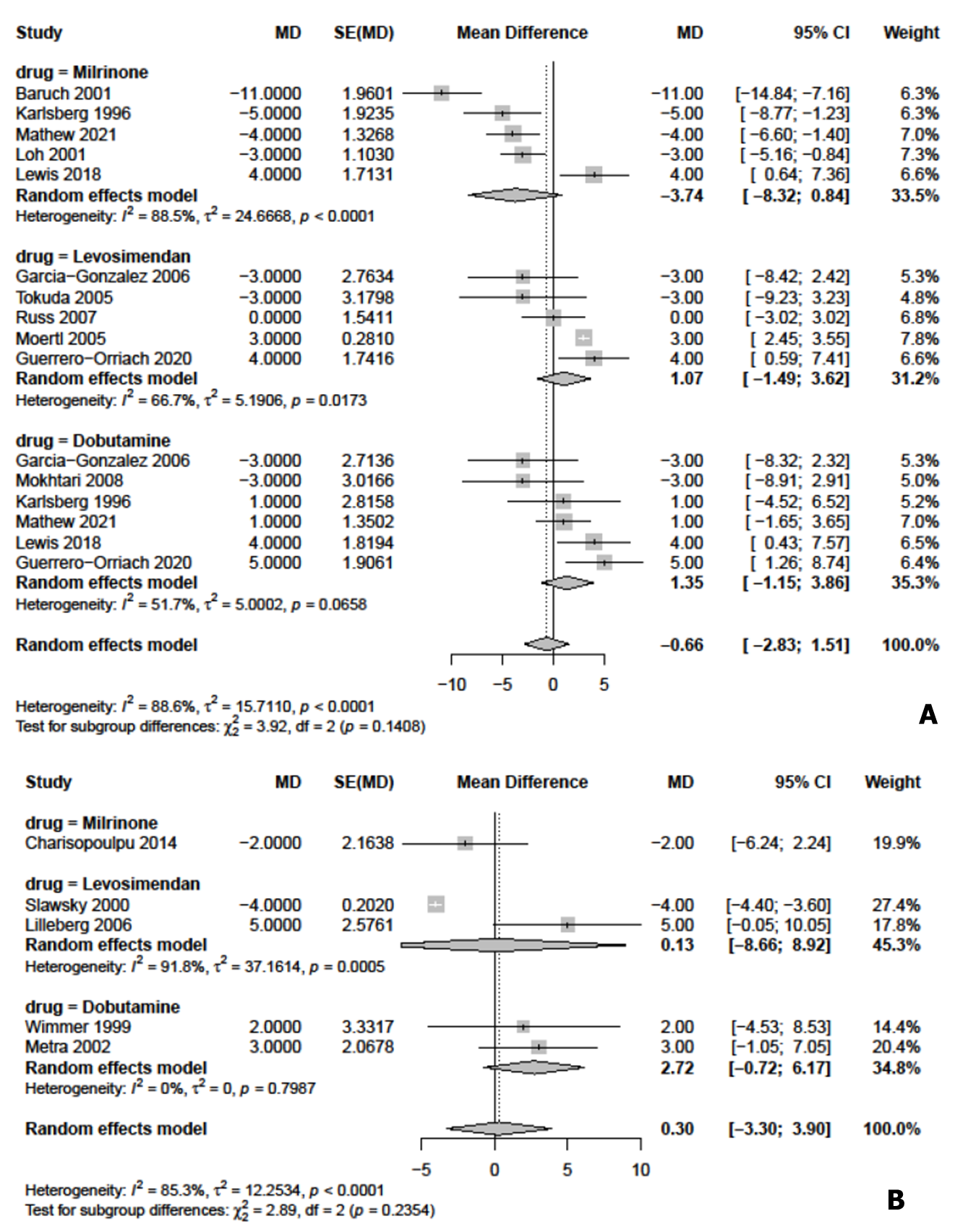


**Supplementary Figure 4**. Mean pulmonary artery pressure changes after the initiation of dobutamine, levosimendan and milrinone analysed according to the specific setting (acute, panel A; chronic, panel B).


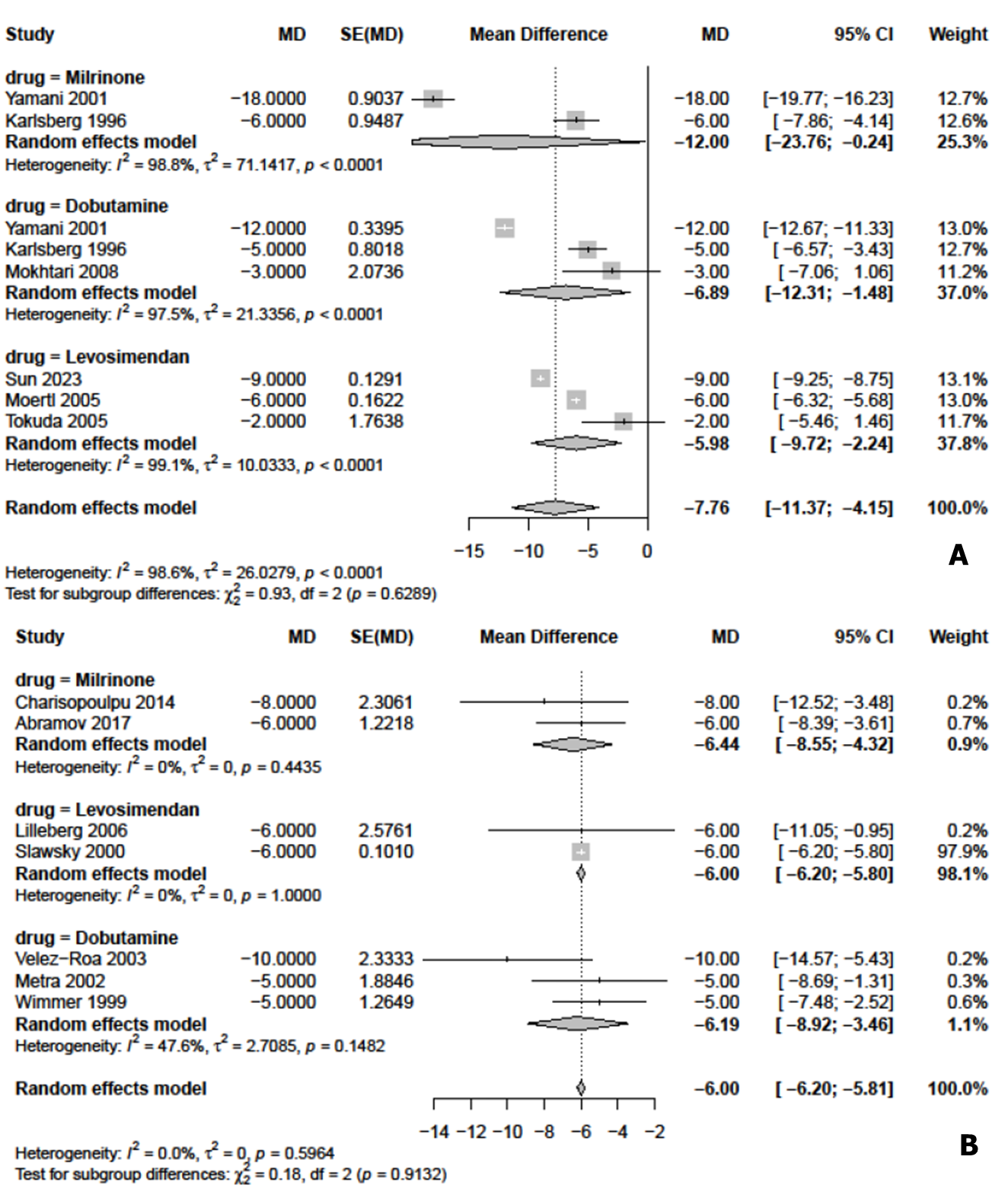


**Supplementary Figure 5**. Pulmonary vascular resistance changes after the initiation of dobutamine, levosimendan and milrinone analysed according to the specific setting (acute, panel A; chronic, panel B).


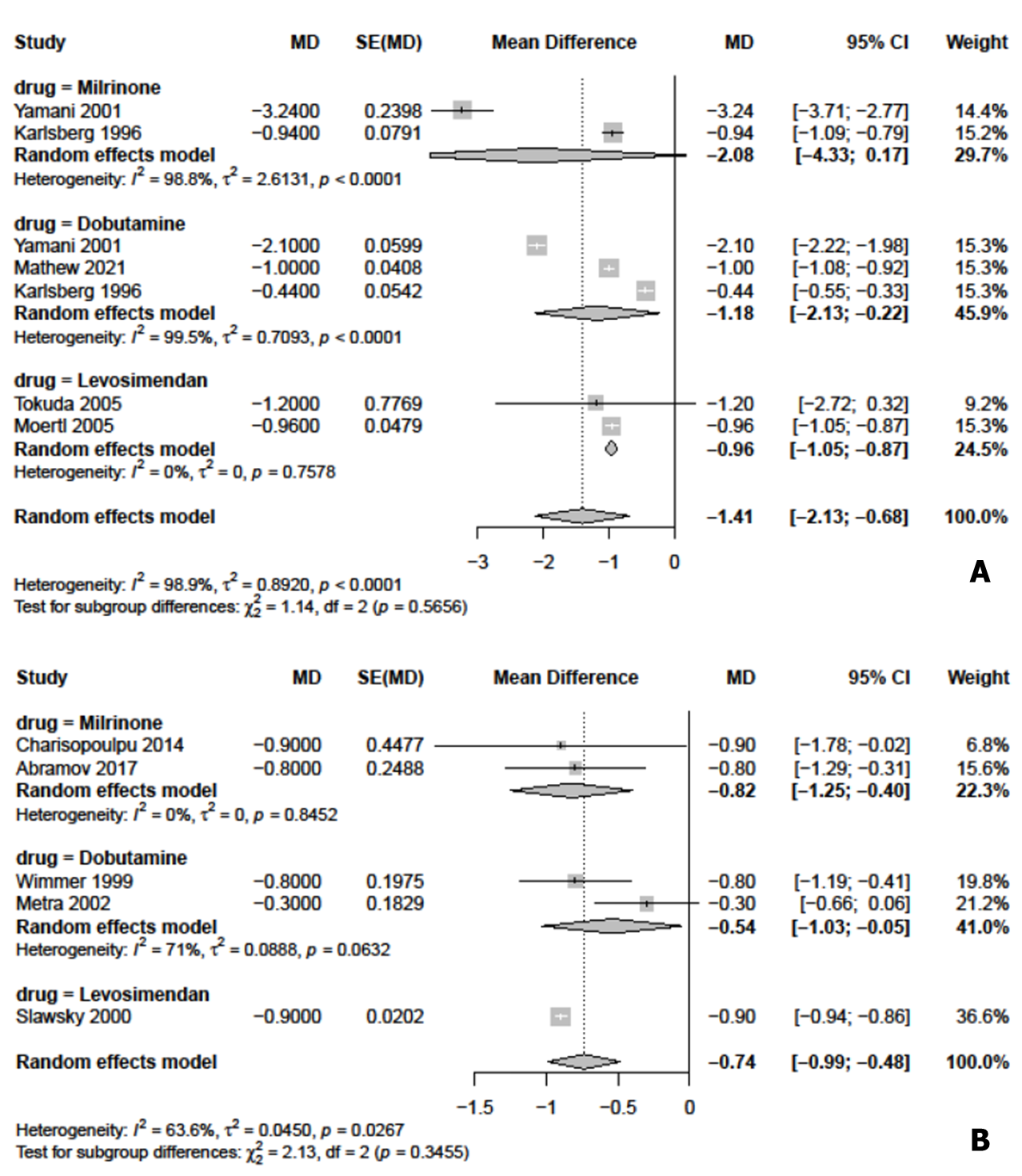


**Supplementary Figure 6**. Systemic vascular resistance changes after the initiation of dobutamine, levosimendan and milrinone analysed according to the specific setting (acute, panel A; chronic, panel B).


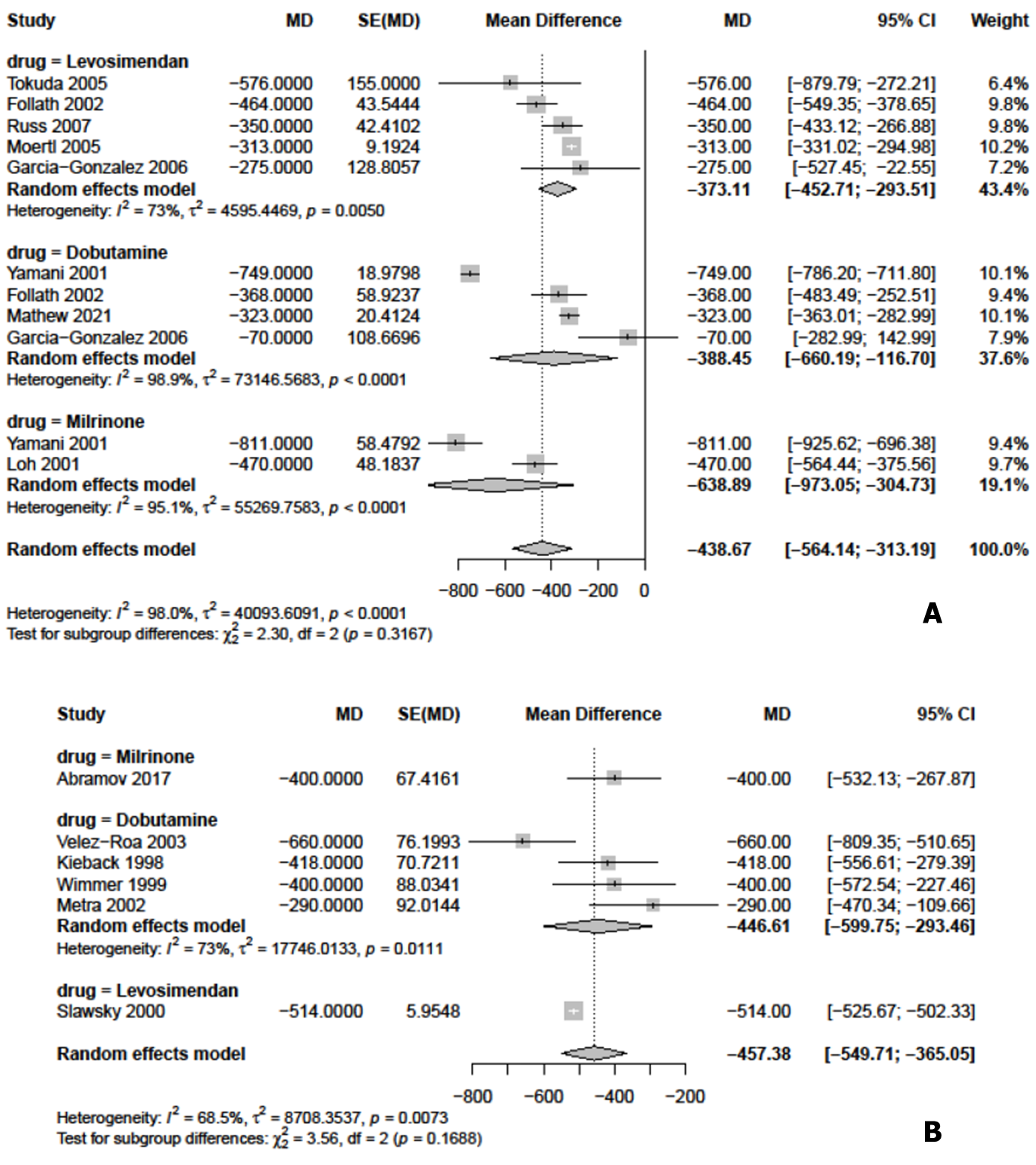


**Supplementary Figure 7**. Funnel plots assessing publication bias for all primary haemodynamic outcomes (CI, MAP, mPAP, PAWP, PVR, and SVR).


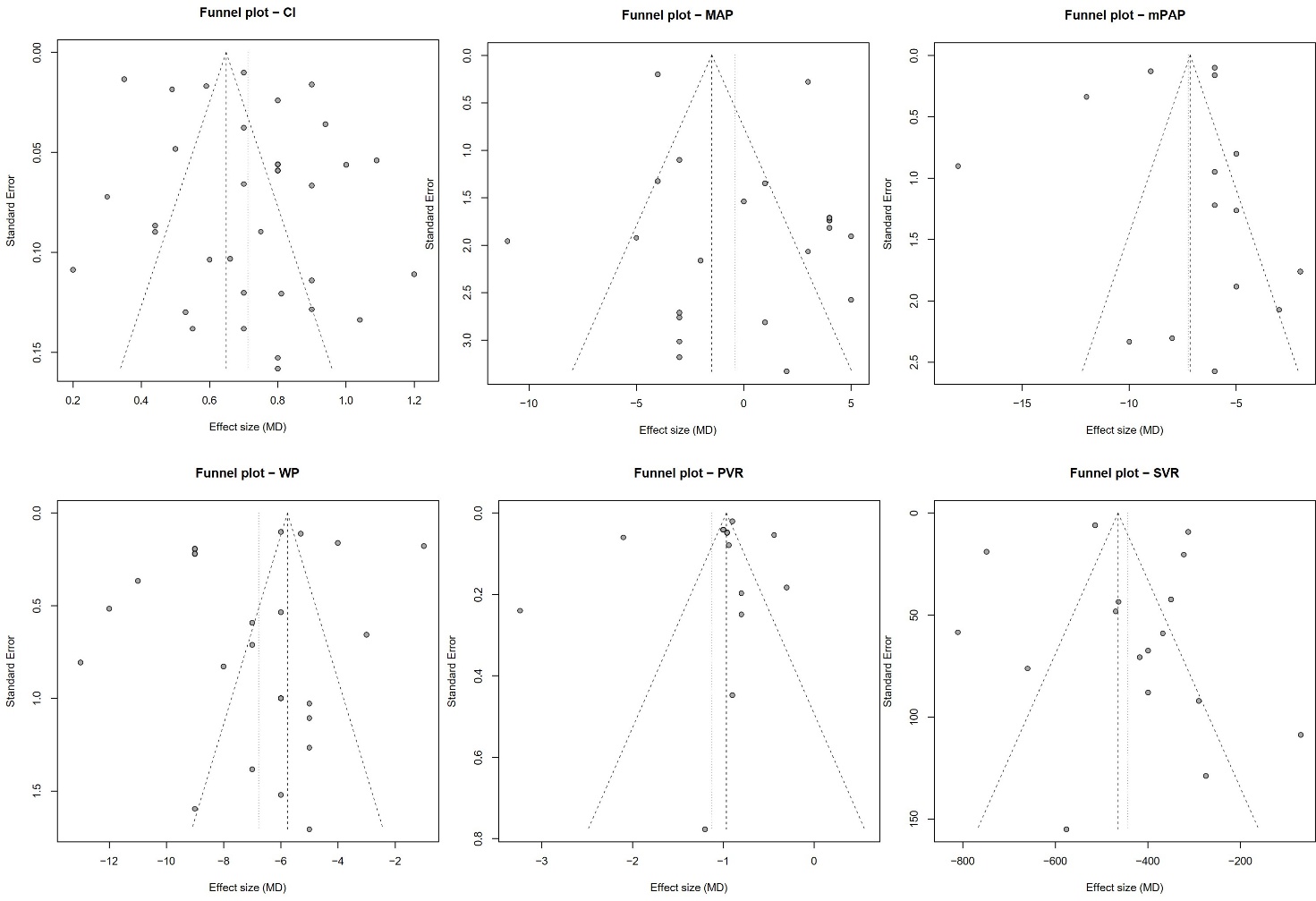

Supplement: xvag120_Supplementary_Data [file xvag120_supplementary_data.docx]
